# Spindle Cell Lipoma of the Soft Palate

**DOI:** 10.1155/2015/813240

**Published:** 2015-03-24

**Authors:** Ahmet Hançer, Can Özbay, Serap Karaarslan, Muzaffer Balaban

**Affiliations:** ^1^Department of Otolaryngology, Şifa University, Bornova, 35100 İzmir, Turkey; ^2^Department of Otolaryngology, Adnan Menderes University, Aytepe Mevkii, 09100 Aydın, Turkey; ^3^Department of Pathology, Şifa University, Bornova, 35100 İzmir, Turkey

## Abstract

Intraoral spindle cell lipomas (SCL) are very rare and comprise ranging between 1.4%–9.8% of all intraoral lipomas. To our knowledge, no case of a SCL located on the soft palate has been reported in the English-language literature. A 31-year-old female was admitted with a swelling in her soft palate. On examination, a 3 cm sessile, nontender swelling was observed on her soft palate. After surgical excision, it was diagnosed as a SCL.

## 1. Introduction

Lipomas are benign tumours or idiopathic proliferation of adipocytes that may contain other mesenchymal tissues and mature adipose tissue arranged in lobules and separated by septa formed of fibrous connective tissue. Clinically, these tumours manifest as asymptomatic, slow-growing submucosal nodules [1, 2]. Approximately 13% of lipomas occur in the head and neck, but oral cavity is unusual site [3]. Oral lipomas account for 2.2 to 4.4% of all benign intraoral tumours with most lesions occurring in the buccal mucosa [3, 4]. The lipomas encountered most commonly are fibrolipomas, osteolipomas, chondrolipomas, angiolipomas, angiomyolipomas, myelolipomas, spindle cell lipomas (SCLs), pleomorphic lipomas, and sialolipomas. SCLs are composed of bland mitotically inactive spindle cells arranged in parallel with the fat cells and associated with thick rope-like collagen bundles [5]. The aetiology and pathogenesis of lipomas remain unclear [6]. Most patients are aged 40 years or older and the tumours are extremely rare in children [6, 7]. In their series of 41 oral cavity lipomas, Juliasse et al. found four (9.8%) SCLs, none of which were located in the soft palate [8]. We present the first case of a SCL located in the soft palate.

## 2. Case Report

**Figure 1:**
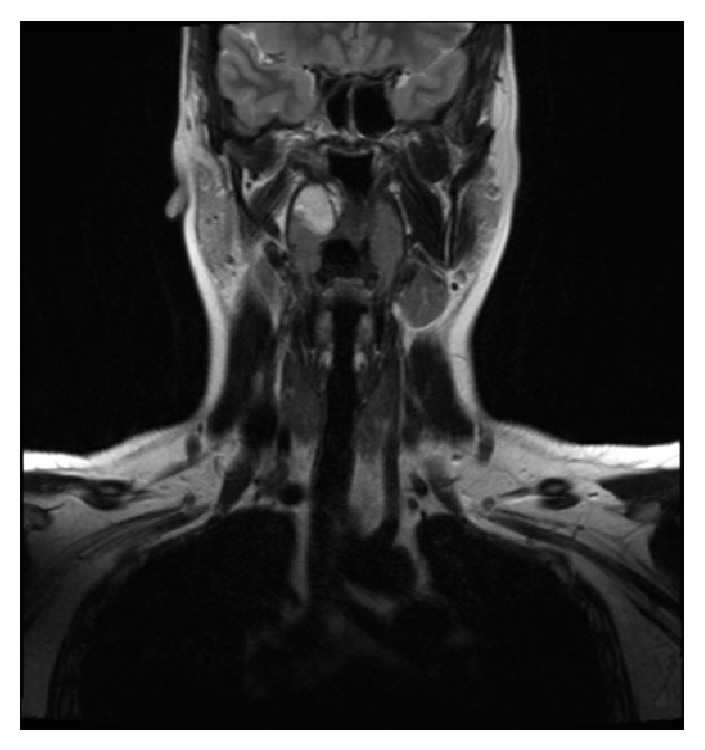
MRI shows a mass in the patient's soft palate.

**Figure 2:**
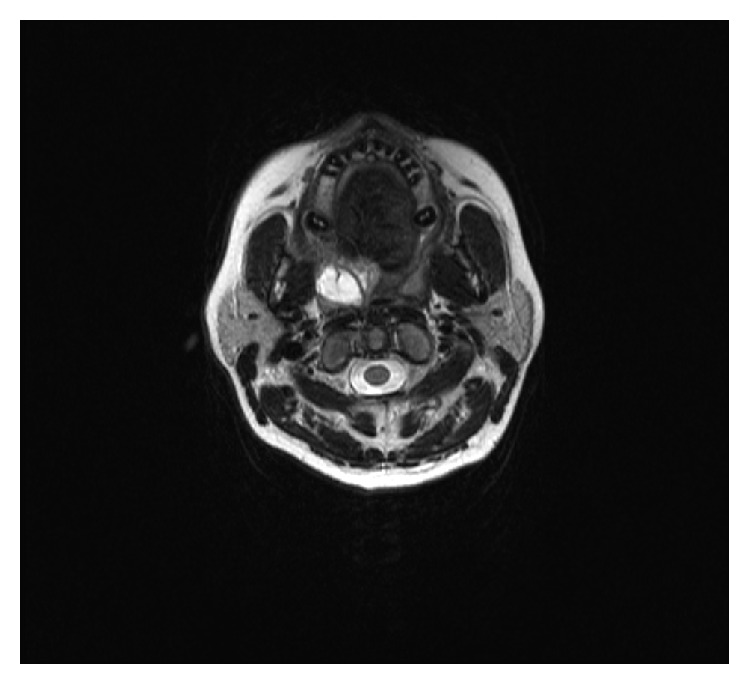
Axial MRI section of the spindle cell lipoma.

A 31-year-old female was admitted with a swelling in her soft palate. On examination, an approximately 3 cm sessile, nontender swelling with normal mucosa and smooth surface was observed on her soft palate. Magnetic resonance imaging (MRI) revealed a 26.5 × 22.5 × 8 mm lump near the right tonsillar palate, which retained contrast substance at its surface and appeared to contain iso- to hypointense fibrils in T1 images and hypointense fibrils in T2 images (Figures 1 and 2).

Based on the initial findings, we thought that this mass might be a cyst of the minor salivary glands, fibroepithelial polyp, benign nerve sheath tumour, or nasopharyngeal tumour. After surgical excision, the specimen was sent to the pathology department. Grossly, the greatest diameter of the mass was 29 mm. The tumour was clearly separated from the surrounding tissues. There was minor salivary gland tissue near the mass.

**Figure 3:**
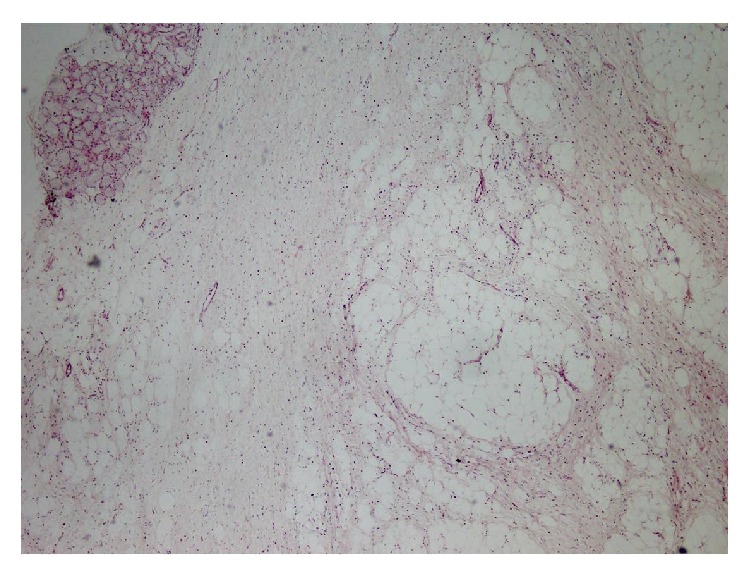
The lesion contained myxoid centres, collagen bands, adipocytes, and spindle cells with a nearby minor salivary gland (H&E ×100).

**Figure 4:**
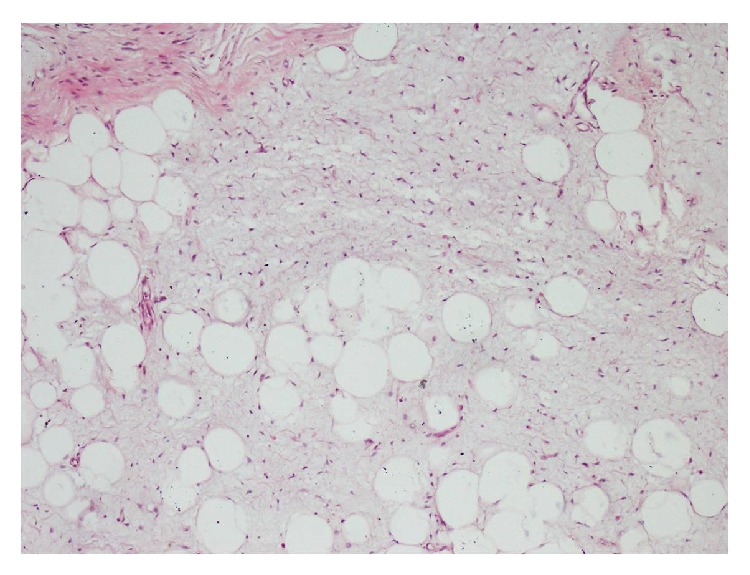
A close-up view of a myxoid area containing adipocytes and spindle cells (H&E ×200).

**Figure 5:**
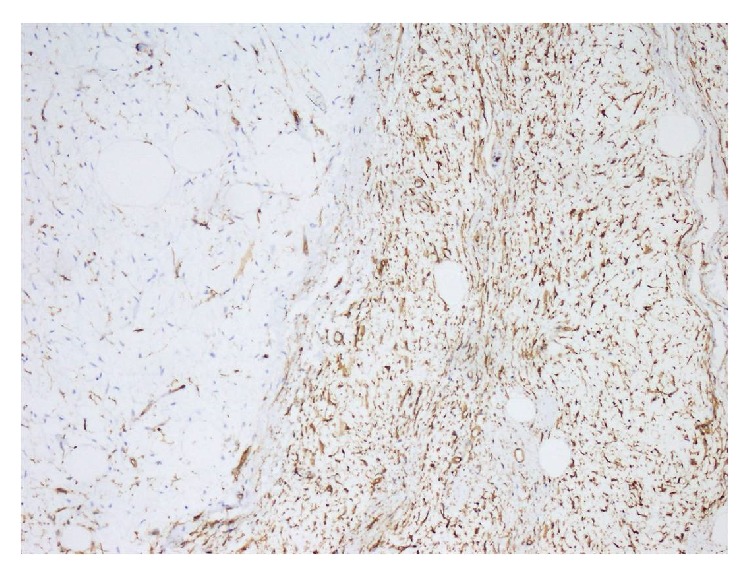
CD34-positive spindle cells (H&E ×200).

Histologically, the mass contained adipocytes and spindle cells and had centres with myxoid character and fine rope-like collagen between these centres (Figures 3 and 4). All immunohistochemical (IHC) staining procedures, including deparaffinisation and antigen retrieval, were performed using the Dako LV-1 Automated Immunostaining System (Dako, Denmark). The mass was strongly positive for CD34 (IR632, FLEX ready to use, Dako, Denmark) (Figure 5) with focal areas that were weakly positive for S-100.

Based on the morphological and IHC findings, the mass was interpreted as a SCL.

## 3. Discussion

Lipomas are benign tumours that develop via the proliferation of adipocytes [9]. There are various theories of the origin of lipomas, including heredity, a hormonal aetiology, infection, metaphase of muscle cells, the presence of lipoblastic embryonic cells, and chronic irritation [10]. Some reports suggest that 13q locus deletions and altered 8q11–13 cause lipomas [11]. Lipomas of the oral cavity are rare and comprise 0.5% of all oral cavity tumours [2]. Lipomas of the oral cavity form a slow-growing mass with a smooth surface [10]. SCLs were first described in 1975 by Enzinger and Harvey [12]. One subtype of SCL that typically presents as a benign lipomatous neoplasm in the posterior neck and back of older males accounts for approximately 1.5% of all lipoma cases [13]. SCLs account for 1.4–9.8% of all intraoral lipomas [2, 8, 14]. Our review of the literature revealed that the tongue and buccal mucosa were the most common sites of intraoral SCLs, which are typically found in males aged 40–70 years. Chandrashekar et al. [10] reported a case and review of the literature that included 26 cases of SCL in 16 males and 10 females between the ages of 29 and 71 years (mean age, 56.5 years). Christopoulos et al. [15] reported first case of SCL located on the hard palate adjacent to the location of the lesion in our case.

Moreover, a review of the clinicopathological features of 35 cases of oral SCL conducted by Manor et al. [16] (two of their cases and 33 from literature) revealed a male : female ratio of 1.92 (23 males and 12 females) in patients between the ages of 23 and 88 years (mean age, 55.0 years). The painless lesions were located on the tongue (*n* = 13), cheek/buccal mucosa (*n* = 11), floor of mouth (*n* = 5), lip (*n* = 2), hard palate (*n* = 2), alveolar ridge (*n* = 1), and maxilla (*n* = 1). Our review of the literature revealed 11 additional cases to those reported by Manor et al. (10 on the tongue and 1 at the mandibular mucogingival junction) [17–20].

The treatment of SCL is surgical removal. After surgery recurrence can occur. Fletcher and Martin-Bates observed one recurrence out of 41 tumours [13].

SCL are rare and the different histological patterns of these lesions might cause diagnostic difficulty. They can be confused with well-differentiated liposarcoma and myxoid liposarcoma. They are differentiated by local myxoid areas that contain CD34-positive spindle cells [13]. Liposarcomas are diagnosed easily by the presence of equal-sized lipocytes separated by fibrous septa and fibrous lipoblasts with hyperchromatic notched nuclei near the fibrous septa. Myxoid liposarcomas show distinct oedema, vessel arborisation, and lipoblasts at the periphery [13]. Angiolipomas and fibrolipomas contain spindle cells and can be confused with SCL. However, angiolipomas are distinguished by vessels at the tumour periphery and fibrolipomas contain dense fibrous tissue bands [9, 13].

Some authors have postulated that spindle cells stem from fibroblasts or are similar to the stellate mesenchymal cells of primitive fat lobules [12, 21, 22]. Others have stated that spindle cells are actually immature mesenchymal cells that remain in position during the transformation to mature lipocytes and are capable of synthesising only collagen at an early stage [13].

As no SCL has been reported in the soft palate in the English-language literature, we decided to share our findings. Although rare, this lesion must be considered when masses are identified at this location.
